# Evaluating a Novel Fly Ash Resin-Reinforced Cement’s Interactions under Acidic, Basic, High-Salinity, and High-Temperature Conditions

**DOI:** 10.3390/polym15163404

**Published:** 2023-08-14

**Authors:** Sherif Fakher, Ali El-Sayed, Layla Sameh, Bassel Abdeltawab

**Affiliations:** Petroleum and Energy Engineering, The American University in Cairo, New Cairo 11835, Egypt

**Keywords:** fly ash, epoxy resin, cementing, oil and gas wells

## Abstract

The ability of cement to withstand harsh conditions is one of its most vital properties, especially in hydrocarbon wells, due to their association with high temperatures, high pressures, acidic components, and erosion. Conventional cement is prone to failure under extreme conditions and is also a costly component in oil and gas wells. This research evaluated the ability of a newly developed cement composed of fly ash reinforced with epoxy resin to withstand the harsh conditions of oil and gas wells. The novel cement was tested for its ability to resist high concentrations of hydrochloric acid (HCl) and sodium hydroxide (NaOH), high salinity, high temperatures, high pressures, gaseous and supercritical carbon dioxide (CO_2_), and crude oil. Results showed that the novel cement had an overall excellent ability to perform under extreme conditions. The performance of the cement was a strong function of the fly ash concentration, with an increase in the fly ash concentration resulting in improvement in the cement. For all tests, the highest degradation for the novel cement that occurred was 0.62% after 7 continuous days of exposure, which is considered an extremely low value. This shows that the novel cement has a strong ability to maintain its integrity under extreme conditions.

## 1. Introduction

Oil and gas well cementing is an integral process during drilling in order to maintain wellbore integrity. Cement serves many functions, including zonal isolation to prevent reservoir fluids’ uncontrollable influx into the wellbore, the preservation of freshwater zones, and the regulation of high-pressure differentials. Based on this, the cement should be able to withstand harsh and severe conditions, including acid attacks, high pressure fluctuations, high temperatures, and erosion and corrosion due to chemicals or solids [1,2,3,4,5,6]. Several types of conventional cement have been developed for different applications based on the conditions mentioned; however, even with the high-performance cement available, cement failures are still a major problem in the oil and gas industry [4,5,6,7]. There is therefore a need for the development of a cement that can withstand the increasingly harsh conditions of the oil and gas wells and also be cost-effective to avoid an increase in the cost of drilling operations.

Conventional oil and gas cement is divided into classes that are labeled alphabetically from A to J. Each of these has unique properties and also different costs based on their applicability ranges. Many researchers have studied the different properties of conventional cement and their failure mechanisms [8,9,10]. Iremonger, S. et al. [11] developed a new apparatus to measure cement integrity through direct strain mapping of the cement sheath under high temperatures for thermal wells. Hart, W. and Smith, T. [12] explained that the key to a successful oil and gas well cementing job is the hydraulic seal in the annulus. This is imperative to ensure no uncontrollable influx of fluids from the formation into the wellbore. Shadravan, A. et al. [13] studied both tensile and shear failures of cement through a laboratory experimentation of cyclic loading on the cement sheath. Their experiments showed that tensile and shear failures become significant at high-temperature and high-pressure conditions. Goodwin, K. and Crook, R. [14] showed that cement sheath failure will occur when there is a high internal casing pressure and is further aggravated by high-temperature conditions. Ding, L. et al. [15] showed that casing failures can occur due to weak or failing cement plugs. These plugs can degrade due to several challenging conditions, such as acidic conditions, high salinity, high temperatures, and high pressure. Meng, M. et al. [16] developed a model that incorporates the cement sheath initial stresses and the transient thermoplastic effects in order to shed light on their significance in cement durability analysis. Pollock, R. et al. [17] emphasized the need for the improvement of cement in thermal stimulation wells due to excessive cement failures under extreme temperature conditions. Kalil, I. et al. [18] used finite element analysis to investigate the casing burst effect when cement is present in the sheath. This was performed to overcome the limitations of previous models that assume that the casing annular space is filled by a fluid equivalent instead of cement. Thiercelin, M. et al. [19] analyzed the mechanical response of set cement in a wellbore to quantify the loss of zonal isolation when the cement bond log response disappears or exhibits unrealistic variations. Forbes, D. and Uswak, G. [20] investigated gas channeling behind the casing, which can result in uncontrollable gas in the wellbore. If this is not mitigated, severe problems can occur on the surface.

One of the additives that has been used extensively in different applications in the oil and gas industry is represented by thermoplastic resins, including epoxide resins. Resins have been used as coating materials for pipes and tubing for thermal insulation due to their low thermal conductivity and high melting points [21,22,23,24]. They have also been used for cement mitigation through squeeze cementing due to the ability to inject them into tight locations easily compared to conventional cement. This is mainly attributed to the lack of solid particles in the resin system and also due to the lack of water in the resin system, therefore avoiding the problem of sedimentation and fluid loss [25,26,27]. One of the most widely used applications for resins is sand coating for proppants. Resin-coated sand has been proven to have superior properties compared to conventional sand. This includes acid resistance, higher compressive strength, uniformness and sphericity, and a higher achievable proppant density [28,29,30,31]. Very little research has focused on using epoxy-based cement and no research has attempted to use a resin in low concentrations as a binding material for fly ash.

Even with the significant advancements in conventional cement, there are still many failures associated with it. This gives rise to the need for cement that is cost-effective and can withstand harsh environments. This research tests a newly developed cement obtained from the combination of a pozzolanic material called fly ash and reinforced with epoxy resin. The cement is tested under severe conditions similar to those that could be encountered in oil and gas wells to determine its ability to resist degradation under these conditions.

## 2. Materials and Methods

The materials, setup, and procedure used to conduct all experiments are explained.

### 2.1. Experimental Materials

The materials used to conduct all the experiments in this research are as follows.

- Fly Ash: The fly ash used was provided by a chemical plant located in Southern Cairo, Egypt. It was provided as a fine powder with a light grey color due to the high aluminosilicate content and low iron oxide content. The fly ash was class F.
- Epoxy Resin: The epoxy resin was commercially available and was provided as a yellow, extremely viscous liquid. The yellow color was due to an added pigment to the resin. The resin had no bisphenol A concentration in it, which made it safe and easy to handle.
- Hardener: For every three parts of resin used, one part of hardener was added. The hardener was provided with the resin as a transparent slightly viscous fluid.
- Graduated Beakers: Beakers were used to weigh the different chemicals and determine their equivalent volumes before mixing. The beakers were made from borosilicate glass and could withstand temperatures up to 250 °C.
- High Accuracy Scale: The scale used had accuracy of four decimal places. This was necessary when weighing the hardener and the epoxy to ensure that the correct mixture was used in all samples.
- Silicon Molds: Spherical molds were used to mold perfect spheres of cement as samples for testing. Spheres were used instead of cubes to account for several experimental setups that could not accommodate a cube and required spherical samples.

### 2.2. Experimental Setup

**Figure 1:**
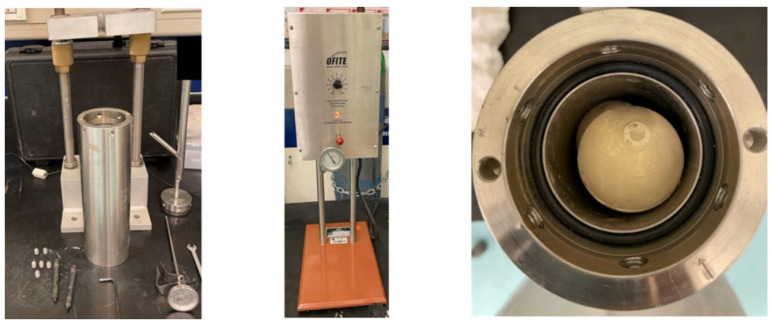
Novel cement samples placed under high pressure.

For all the experiments conducted, the samples were placed in a container with the fluid of interest for the duration of the experiment. The setup used to conduct the high-pressure CO_2_ experiments is presented in Figure 1. The setup was composed of a high-pressure vessel that was connected to a heating element. The samples were placed in the vessel and then the vessel was sealed. The vessel was connected to a high-pressure CO_2_ cylinder to provide the pressurized CO_2_ [32,33,34,35,36,37].

### 2.3. Experimental Procedure

The procedure followed to conduct all the experiments was as follows.

- Prepare the cement samples and leave them to cure overnight until fully set. Recover the samples and weigh them before running any experiment.
- Prepare the experimental solution in a transparent container and ensure that the volume of the solution prepared is sufficient to fully cover the cement sample. Place the cement sample in the container and begin the experiment.
- Leave the cement sample in the container for 7 consecutive days. Visually examine the sample every 6 h and weigh the sample every 24 h.
- Record the change in the weight of the sample every 24 h and plot the results after 7 days. Determine the weight increase or the weight decrease percentage with time to evaluate sample degradation.

### 2.4. Novel Cement Synthesis

The procedure followed to synthesize the cement samples was as follows.

a. Weigh the pre-calculated mass of the chemicals, including the epoxy resin, the hardener, and the fly ash, and store each in a separate container.
b. Pour the resin into a large plastic container. Ensure that all the resin has been displaced. This is done by leaving the resin to pour overnight while occasionally displacing it with a spatula.
c. Place a small volume of fly ash in the resin and mix by hand. This is to avoid the formation of lumps in the slurry. The sample is constantly mixed until all fly ash is added. This takes two to six hours depending on the volume of fly ash. An electric blender is used initially to heat up the sample, which impacts the resin. Moreover, when a large mass of resin is added, the blender can no longer blend the resin–fly ash mixture efficiently.
d. Once the resin–fly ash sample is well mixed and stable, the hardener is added while vigorously mixing all three components. It is important to note that once the hardener is added, the cement setting will begin. It is therefore important to quickly set the cement slurry in the mold before the slurry becomes too difficult to pour. This will usually happen 30 min to 1 h after the hardener is added, depending on the slurry formulation.
e. Once the slurry is set in the mold, it is left to fully cure and set overnight for at least 12 h. The samples are then retrieved and visually inspected for air pockets or heterogeneities in the sample. If the sample has any heterogeneities or air pockets, it is discarded. The samples are then labeled and prepared for experimentation.

The cement characterization was conducted to measure its density for workability and also the sedimentation behavior of the fly ash. The density results are presented in Table 1. Based on the results, the 70 wt% fly ash had a much higher density compared to the other samples. Moreover, when compared to the conventional cement, the 70% fly ash sample was higher, which indicated poor workability. The 50 wt% fly ash sample had a lower density compared to conventional cement and was therefore used as the maximum weight percent fly ash in the novel cement slurry. For all samples, no sedimentation occurred, which indicated that the fly ash was stable in the slurry.

**Figure 2:**
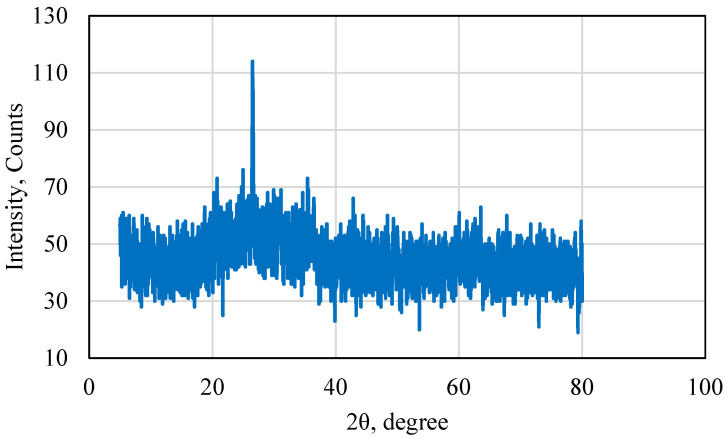
X-ray diffraction results for fly ash sample used.

X-ray diffraction (XRD) was performed on the fly ash used in this research to test its properties. The XRD result is presented in Figure 2. Based on the analysis of the XRD results, it was found that the sample used was rich in aluminosilicates and low in iron oxides. This was highly advantageous since the aluminosilicates allowed the fly ash particles to form strong bonds, which enhanced the strength of the samples. Low metal content is also advantageous since it prevents cement corrosion and also reduces interference with some tools that are sensitive to metals. Based on the XRD, the fly ash used in this research could be classified as type F.

## 3. Results and Analysis

The performance of the novel cement with an acid, base, high-salinity solution, high-temperature conditions, high-pressure carbon dioxide, crude oil, and acetone is presented and explained. For several experiments, two samples of each fly ash concentration were tested for repeatability. The impact of all the factors on the cement was tested based on the imbibition of the different fluids in the cement lattice, which is the main mechanism by which different fluids will degrade cement in real field applications.

### 3.1. Base Experiment—Distilled Water

**Figure 3:**
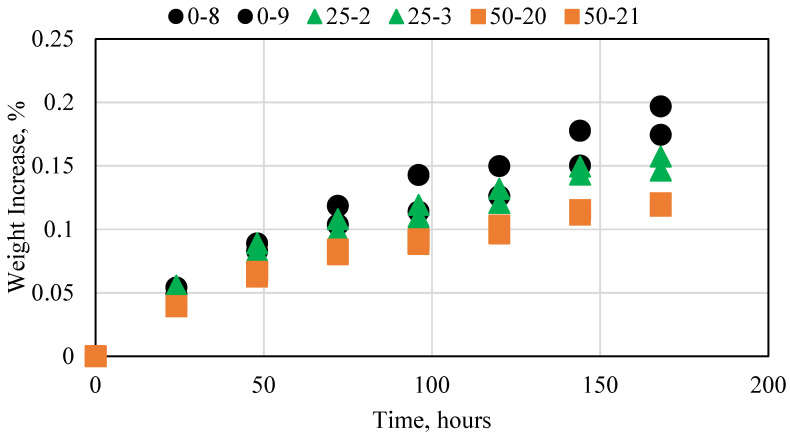
Novel cement interaction with water at ambient conditions.

A base experiment was conducted in distilled water to act as a reference for all other experiments conducted. Figure 3 shows the results for the 0, 25, and 50 wt% fly ash cement sample experiments. All experiments were conducted at atmospheric pressure and ambient temperature conditions. The assessment criteria for all samples were the variation in weight. If the weight increases, then the fluid is imbibing into the cement sample, which impacts its integrity. The smaller the weight variation, the better the result. All three cement samples exhibited a small increase in weight every day. The samples with a higher fly ash concentration resulted in a smaller decrease. This was due to the fly ash that caused the fluid to imbibe less in the sample. Overall, however, the change was extremely small and thus had no impact on any of the samples. The weight change was less than 0.001 g for each sample. This base case was used as the comparison for all the experiments conducted in this research.

### 3.2. Cement Interaction with Acid

**Figure 4:**
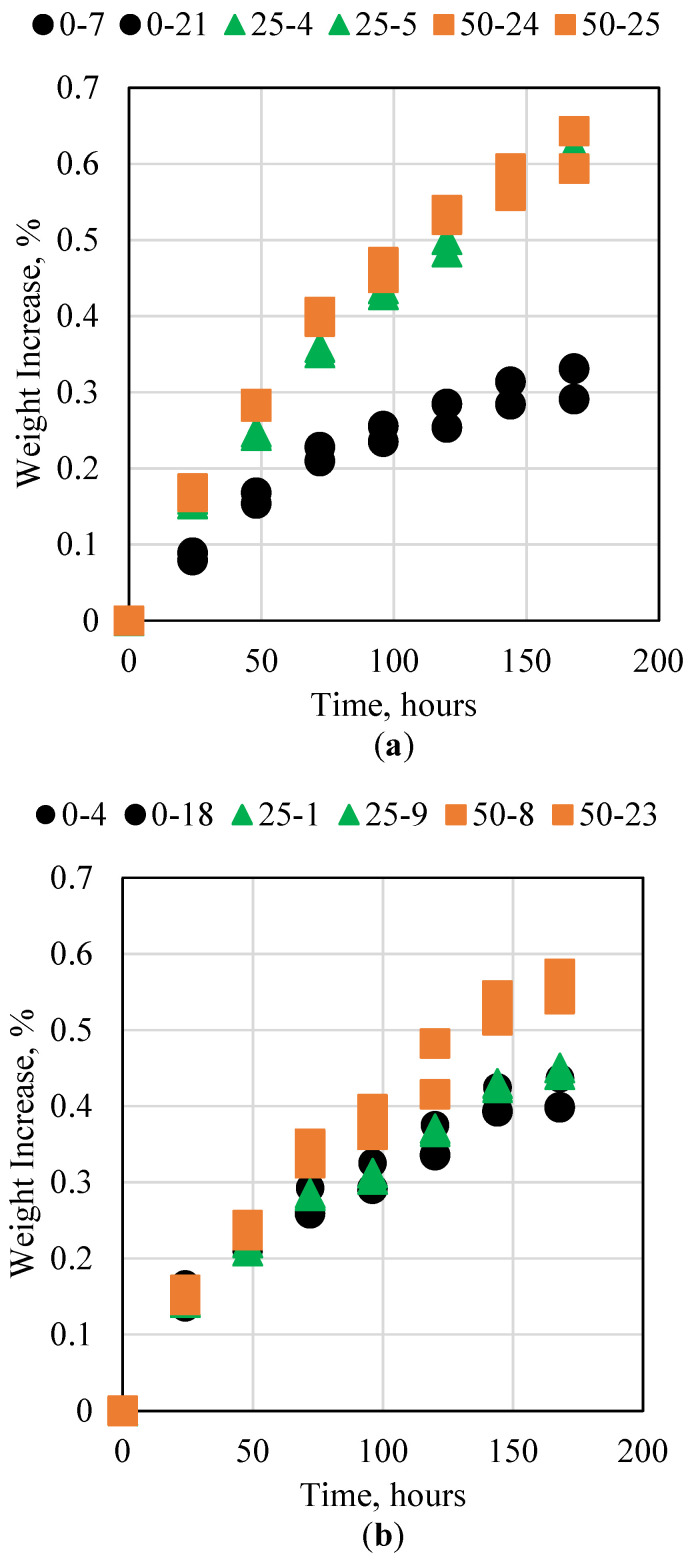
Novel cement interaction with (**a**) 15% and (**b**) 28% HCl at ambient conditions.

The cement in oil and gas wells is subjected to acidic conditions in many cases. The acid can come directly from the formation in the form of acidic oil or hydrogen sulfide. In both cases, conventional cement can fail rapidly, especially if a low-grade cement is used. Acid can also be injected into oil and gas wells during matrix acidizing or acid fracturing. In this case, a wide variety of acids are used, ranging from acetic acid to extremely potent acids such as hydrochloric acid and hydrofluoric acid. This research investigated the resistance of the cement to 15% HCl and 28% HCl, as 15% HCl is commonly used in well stimulation applications, whereas 28% is considered an extreme value but was still tested to evaluate the degree of the novel cement’s integrity. Figure 4 shows the weight increase percentages for the 0%, 25%, and 50% by weight fly ash cement samples placed in the acid solution for 7 consecutive days. For the 15% HCl test, all the samples exhibited a weight increase of less than 1%, which indicates strong resistance to acid. The samples with fly ash exhibited larger swelling compared to the samples with no fly ash, which indicates a negative impact. When the acid concentration was increased to 28%, however, the opposite trend was observed, with the samples containing fly ash showing a smaller weight increase, whereas the sample with no fly ash degraded more. This could be due to the sample with fly ash reaching the maximum fluid imbibition and therefore it could not be impacted beyond 0.66%. Moreover, the pH of the acid was measured at the beginning and end of the experiments. The pH remained the same, which is a strong indication that no reaction occurred between the cement samples and the acid and therefore there was no alteration in pH.

### 3.3. Cement Interaction with Base

**Figure 5:**
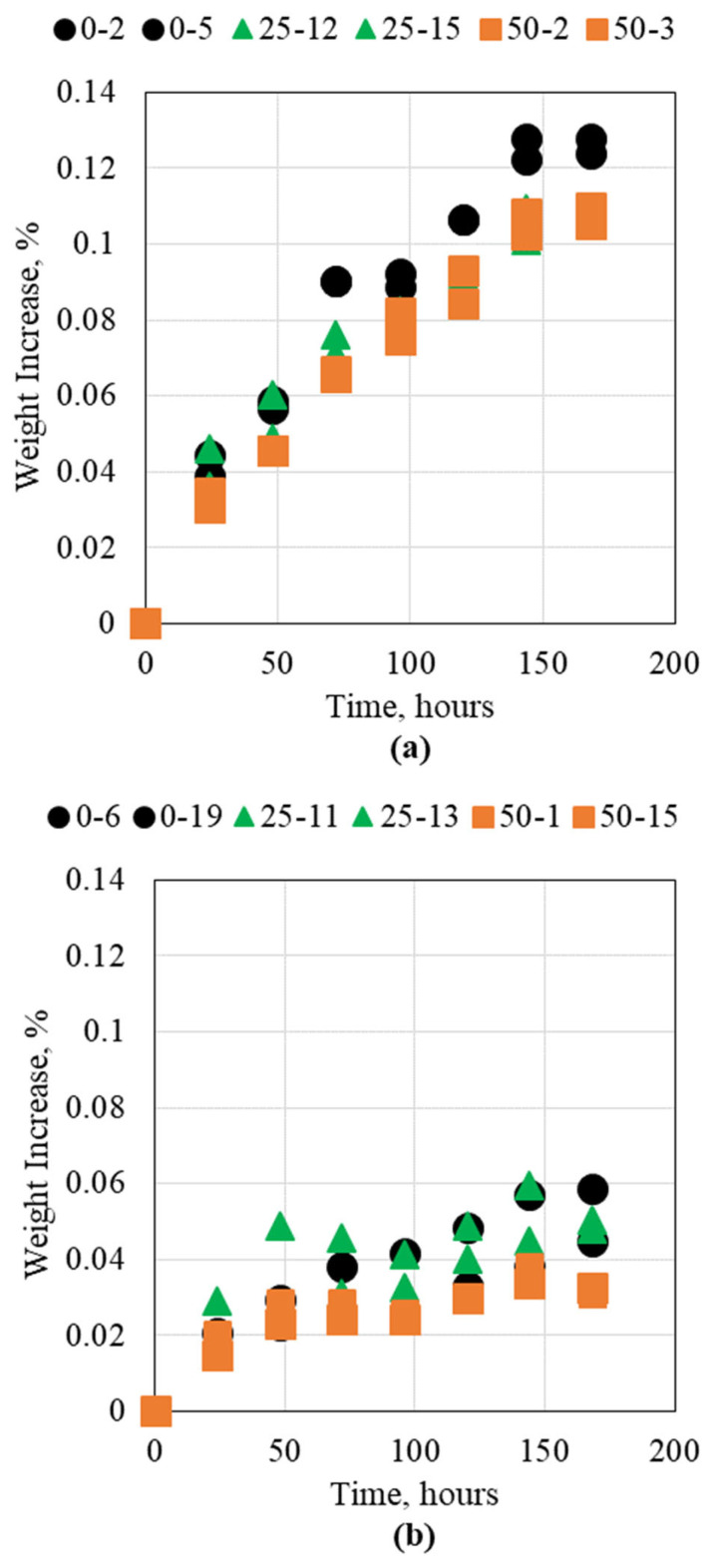
Novel cement interaction with (**a**) 15% and (**b**) 28% NaOH at ambient conditions.

The alkaline conditions in oil and gas reservoirs can be manifested in two main fluids, including alkaline flooding for enhanced oil recovery and high-pH water injection. The main alkaline chemicals used in these processes are sodium hydroxide (NaOH) and sodium bicarbonate. These chemicals can interact with the cement and alter its properties. This research studied the impact of the cement to avoid degradation when placed in an alkaline solution of 15% NaOH and 28% NaOH for 7 consecutive days. The results for the 0%, 25%, and 50% fly ash samples are presented in Figure 5. For both NaOH concentrations, all three samples were not impacted significantly. Moreover, it was observed that no significant variation occurred when fly ash was added to the sample. This indicates that neither the fly ash nor the resin was impacted by the alkaline solution. This in turn shows that the novel cement is not impacted by alkaline conditions and can therefore be used safely during alkaline flooding or in high-pH water conditions.

### 3.4. Cement Interaction with High Salinity

**Figure 6:**
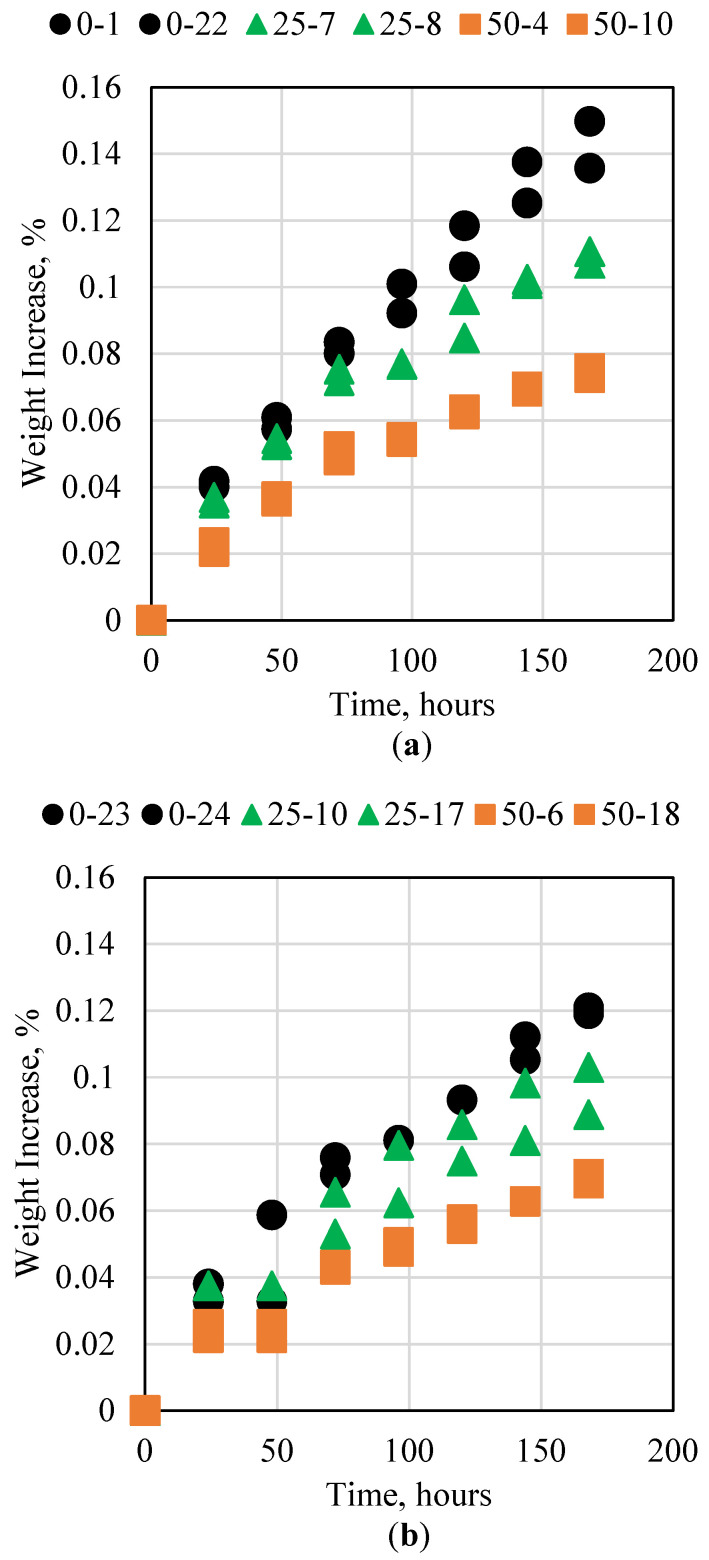
Novel cement interaction with (**a**) 15% and (**b**) 20% NaCl solution at ambient conditions.

Formation water associated with oil and gas reservoirs usually has extremely high salinity. This can exceed 150,000 ppm or 15 wt%; for comparison, the salinity of seawater is approximately 40,000 ppm or 4 wt%. Due to its high salinity, formation water can be extremely corrosive and thus can damage the cement. If the high-salinity water damages the cement, it can then form contacts with the metal tubing that maintains the well integrity and corrode it as well, which can compromise the integrity of the entire wellbore. This research therefore investigated the impact of high-salinity brine, using 15% and 20%, on the novel cement. Figure 6 shows the results for the 0%, 25%, and 50% fly ash samples. Based on the results, none of the novel cement samples was impacted significantly by the brine, even at high NaCl concentrations. The sample with 0% fly ash appeared to have the largest impact, whereas increasing the fly ash concentration reduced the weight change slightly. This could be attributed to the reduction in available pores due to the fly ash occupying them. Overall, the results show that the novel fly ash resin cement has good resistance to high-salinity conditions.

### 3.5. Cement Interaction with High-Temperature Water

**Figure 7:**
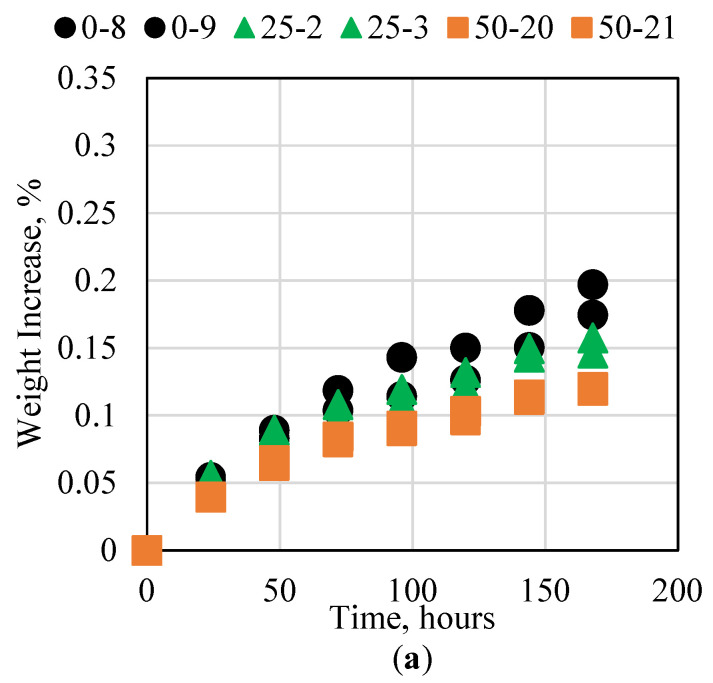

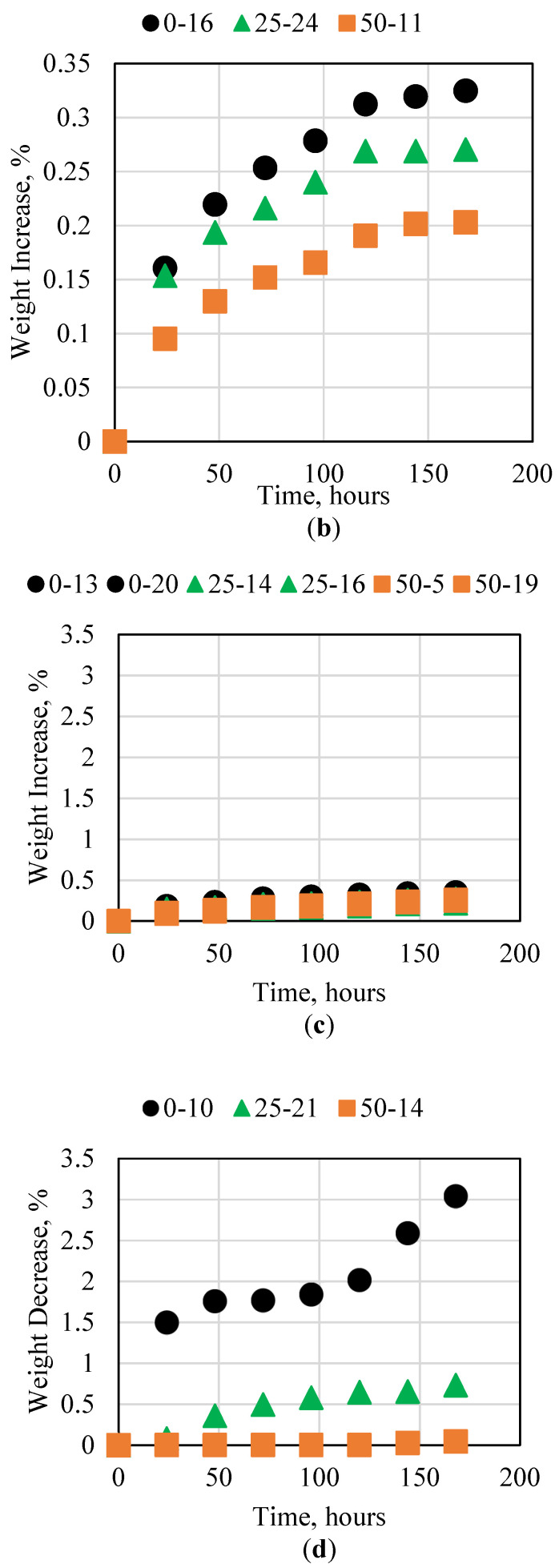
Novel cement interaction with DI water at (**a**) 25 and (**b**) 40 °C, (**c**) 60 °C, and (**d**) 100 °C.

Different oil and gas reservoirs exist at different depths and thus usually have different temperatures. Although the temperature of the reservoir itself does not vary significantly, conventional cement is usually applicable at temperatures less than 60 °C unless additives are included, or a special temperature-resistant cement is used, which is usually very high in cost. The ability of the novel cement to withstand temperatures up to 100 °C for seven consecutive days was tested in this research. The results for the 25 and 40 °C experiments are presented in Figure 7. For the room-temperature experiment, all three cement samples had an overall weight increase of less than 0.2%, with slight variation between the samples. When the temperature was increased to 40 °C, the sample with no fly ash, 100% resin, exhibited a larger weight increase compared to the other samples with different concentrations of fly ash. It is important to note that the fly ash did not reduce the overall thermal conductivity of the cement. When the thermal conductivity of the epoxy resin was measured, it was found to be 0.158 W/mK, whereas the thermal conductivity of the fly ash was 1.41 W/mK; therefore, the fly ash could absorb more heat than the resin. The mechanism by which the fly ash reduced the overall thermal degradation of the sample was by filling the available void spaces in the sample, thus preventing the water from imbibing in the sample. This in turn reduced the overall contact surface area between the water and the cement sample, thus reducing the thermal conductivity. The samples exhibited very little degradation at 60 °C. This shows that the novel cement can function well at these conditions. When the temperature was increased to 100 °C, however, several interesting results were observed. The sample with no fly ash began to degrade significantly, especially after four days. The samples with fly ash exhibited less than 1% degradation, with the increase in fly ash resulting in less degradation. Moreover, the experiments conducted at 100 °C were the only experiments that showed a weight decrease rather than an increase. This was investigated further and we found that the samples lost weight due to particles of the epoxy melting and separating from the sample. Some of the weight loss was offset by water imbibition; however, the overall weight increase was still noticeable.

### 3.6. Cement Interaction with Carbon Dioxide

**Figure 8:**
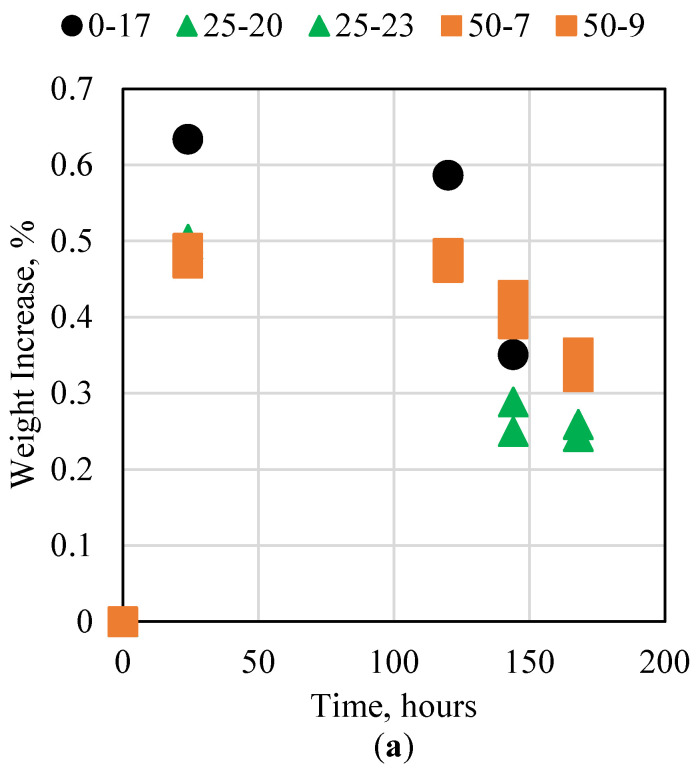

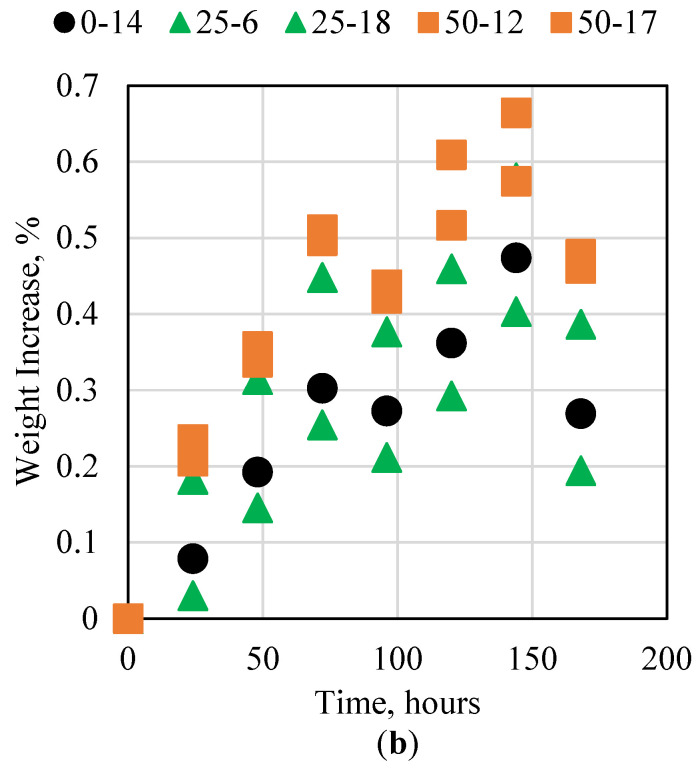
Novel cement interaction with CO_2_ at 60 °C and (**a**) 500 psi and (**b**) 1200 psi.

CO_2_ injection in oil and gas reservoirs has been increasing globally in recent years, especially for CO_2_ storage, under the carbon capture, utilization, and storage initiative. Since CO_2_ is acidic, it reacts with conventional cement significantly due to the high pH of conventional cement. This reaction can result in cement’s degradation with time, which can compromise the ability to store CO_2_ in depleted oil and gas reservoirs for long durations. The impact of CO_2_ on the novel cement was therefore studied in this research. The two pressures investigated were 500 and 1200 psi, both at 60 °C. At 500 psi and 60 °C, the CO_2_ is in the gaseous phase, whereas at 1200 psi and 60 °C, the CO_2_ is in the supercritical phase; therefore, this allowed us to study both phases of CO_2_. The samples were loaded in a high-pressure vessel and remained in contact with CO_2_ for 7 consecutive days. The weight of the sample was measured daily by depressurizing the vessel, weighing the sample, and then repressurizing to the same conditions. The impact of CO2 on the 0, 25, and 50 wt% fly ash samples under both pressure conditions is shown in Figure 8. The most significant observations in the CO_2_ experiments were the skewness of the data points and the opposing trends between the two experiments. When the samples were pressurized at 500 psi, as time progressed, the weight increase of the samples decreased. The opposite was observed in the 1200 psi experiments, where the weights of the samples increased with time. This could be explained in terms of the CO_2_ phase. At 500 psi, the CO_2_ was in the gaseous phase; therefore, it imbibed in the pores of the novel cement sample quickly. As time progressed, however, the CO_2_ was expelled from the sample due to epoxy swelling, which filled the voids. When the 1200 psi pressure was used, the CO_2_ was in the supercritical phase. In this phase, the CO_2_ became denser, which in turn limited its ability to imbibe into the cement sample; therefore, the imbibition process took more time. In both cases, however, the imbibition was forced, and the CO_2_ caused a weight increase of less than 1%, which indicated a minimal impact on the integrity of the cement sample.

### 3.7. Cement Interaction with Crude Oil

**Figure 9:**
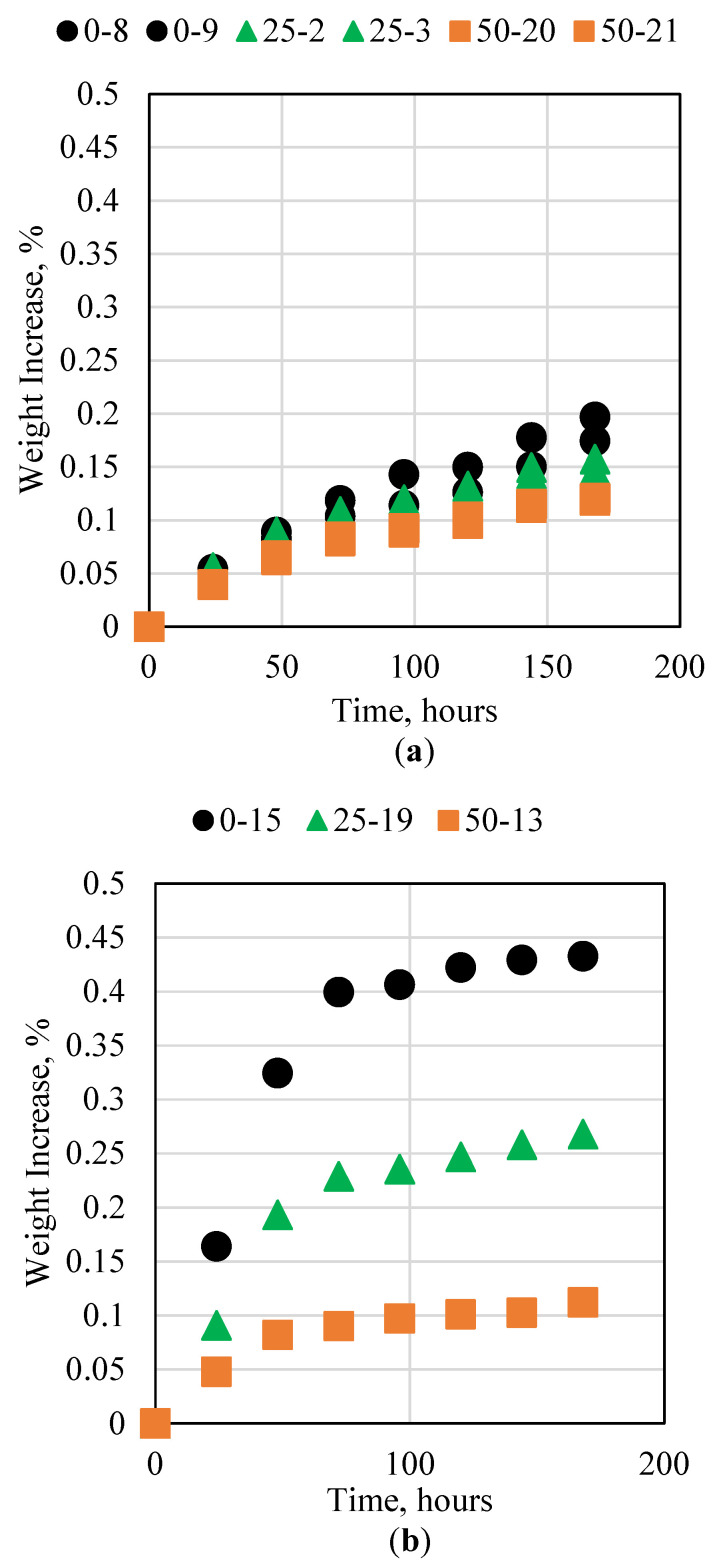
Novel cement interaction with (**a**) DI water and (**b**) crude oil at 25 °C.

After setting in the oil and gas reservoirs, the cement acts as a barrier for reservoir fluids to prevent them from invading the wellbore in an uncontrolled manner, which can result in accidents on the surface. The reservoir fluids that the cement is usually in contact with include high-salinity brine, acidic gases such as hydrogen sulfide, and crude oil. It was therefore important to assess the performance of the cement when in contact with all three fluids. The experiments presented in Figure 9 show the interaction of the novel cement with crude oil obtained from an oil field in the Western Desert of Egypt, and the figure compares the performance of the cement to samples placed in distilled water. Based on the results, the samples appeared to be impacted more by the crude oil than the distilled water, with the weight increase being slightly larger. This could have been due to the acidic nature of the crude oil compared to the neutral conditions of the water. Based on the previous experiments, the acid had a slightly larger impact on the cement samples compared to neutral conditions, which was the same as the observation for the crude oil experiments. The sample with no fly ash had the highest impact, whereas the increase in the fly ash concentration resulted in a decrease in the weight change. Overall, however, the impact of the crude oil on all the samples was less than 0.5%, which is extremely small.

### 3.8. Cement Interaction with Acetone

**Figure 10:**
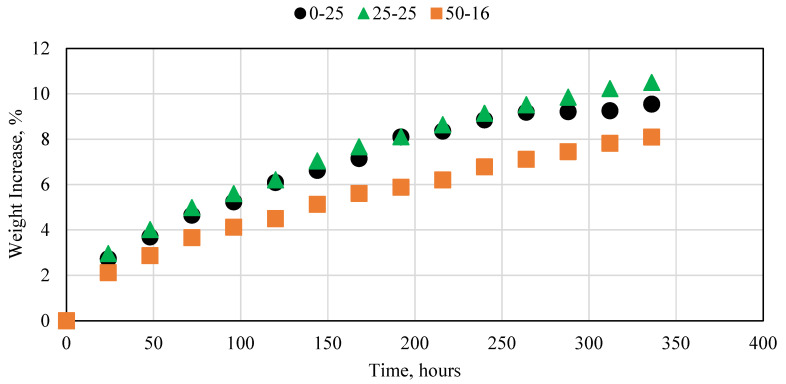
Novel cement interaction with acetone at ambient conditions.

One of the most important operations in the oil and gas industry is the ability to mitigate cement problems or errors when needed. To achieve this, the cement must be able to be improved or removed after setting. This is a large challenge for conventional cement since it cannot be modified significantly after setting. The ability to remodel the novel cement after setting was investigated in this research. Epoxy resins have been proven to be significantly impacted by ketones; therefore, acetone was used to alter the properties of the novel fly ash resin cement. The cement samples were submerged in acetone for 14 consecutive days and their weight was measured daily. The impact of acetone on the 0, 25, and 50 wt% fly ash samples is presented in Figure 10. For all three samples, the weight change was significantly high, compared to all the previous experiments. This indicated that the acetone was imbibed in the sample in a rapid manner. The samples were visually inspected, and it was observed that the samples’ diameters increased significantly. Following this, the samples began to crack. When the samples were removed from the acetone, they began to crumble, and their outer layer was destroyed. This indicates that acetone can be used as an effective chemical to mitigate cement problems or for cement removal when using the novel fly ash resin cement in real field applications. This is a huge advantage over conventional cement, which does not have this property.

## 4. Discussion

A summary of all the experiments conducted in this research is presented in Table 2. Based on all the results, for all the experiments excluding the acetone cement degradation test, the weight percent increase or decrease for the samples containing fly ash was less than 1%. This indicates that the novel cement had significant tolerance to the severe conditions of the oil and gas wells.

## 5. Conclusions

This research studied the ability of a novel fly ash-based and resin-reinforced cement to resist degradation under the extreme conditions of oil and gas wells. The cement was tested under acidic, basic, high-temperature and -pressure, and high-salinity conditions. Future experiments will include the compressive strength measurement of all the samples tested in this research. The main conclusions obtained from this research are as follows.

- The novel fly ash epoxy cement was found to have strong resistance to acidic and basic conditions. It also performed significantly well under high-salinity conditions and when subjected to high temperatures.
- At 100 °C, the novel fly ash epoxy cement lost weight, compared to all other experiments, where the samples gained weight. This was due to the resin melting, which resulted in the loss of small parts of the novel cement sample.
- The novel cement managed to withstand the high-pressure CO_2_ conditions with minimal degradation. This indicates the ability to use the cement for CO_2_ storage applications.
- The novel cement can be easily removed or mitigated after placement and setting in oil and gas wells using acetone. This is a huge advantage over conventional cement.

## Figures and Tables

**Table 1:** Novel cement density for different fly ash concentrations.

Fly Ash Concentration, wt%	Density, kg/m^3^
0	1330
25	1450
50	1700
70	1980

**Table 2:** Impacts of different parameters on novel cement.

Parameter	Sample	Temperature, °C	Pressure, psi	Sample Change	Impact
DI Water	0% FA	25	14.7	Increase	0.184
	25% FA				0.146
	50% FA				0.118
15% HCl	0% FA	25	14.7	Increase	0.31
	25% FA				0.6
	50% FA				0.62
28% HCl	0% FA	25	14.7	Increase	0.42
	25% FA				0.45
	50% FA				0.55
15% NaOH	0% FA	25	14.7	Increase	0.126
	25% FA				0.105
	50% FA				0.105
28% NaOH	0% FA	25	14.7	Increase	0.06
	25% FA				0.05
	50% FA				0.03
15% NaCl	0% FA	25	14.7	Increase	0.14
	25% FA				0.11
	50% FA				0.08
20% NaCl	0% FA	25	14.7	Increase	0.12
	25% FA				0.08
	50% FA				0.07
DI Water 40 °C	0% FA	40	14.7	Increase	0.33
	25% FA				0.27
	50% FA				0.20
DI Water 60 °C	0% FA	60	14.7	Increase	0.33
	25% FA				0.25
	50% FA				0.24
DI Water 100 °C	0% FA	100	14.7	Decrease	3
	25% FA				0.73
	50% FA				0.04
CO_2_ 500 psi	0% FA	60	500	Increase	0.35
	25% FA				0.26
	50% FA				0.32
CO_2_ 1000 psi	0% FA	60	1000	Increase	0.47
	25% FA				0.38
	50% FA				0.45
Crude Oil	0% FA	25	14.7	Increase	0.43
	25% FA				0.27
	50% FA				0.112
Acetone	0% FA	25	14.7	Increase	9.5
	25% FA				10.5
	50% FA				8.1

## Data Availability

Not applicable.
